# Entomological Survey Confirms Changes in Mosquito Composition and Abundance in Senegal and Reveals Discrepancies among Results by Different Host-Seeking Female Traps

**DOI:** 10.3390/insects12080692

**Published:** 2021-07-31

**Authors:** El Hadji Malick Ngom, Chiara Virgillito, Mattia Manica, Roberto Rosà, Verena Pichler, Noemi Sarleti, Isseu Kassé, Mawlouth Diallo, Alessandra della Torre, Ibrahima Dia, Beniamino Caputo

**Affiliations:** 1 Medical Zoology Pole, Institut Pasteur de Dakar, Dakar 12500, Senegal; ElHadjiMalick.NGOM@pasteur.sn (E.H.M.N.); bijoukasse@gmail.com (I.K.); Mawlouth.DIALLO@pasteur.sn (M.D.); 2 Department of Public Health and Infectious Diseases, Sapienza University of Rome, 00185 Rome, Italy; chiara.virgillito@uniroma1.it (C.V.); verena.pichler@uniroma1.it (V.P.); sarleti.1606633@studenti.uniroma1.it (N.S.); alessandra.Dellatorre@uniroma1.it (A.d.T.); 3 Department of Biodiversity and Molecular Ecology, Edmund Mach Foundation, 38098 San Michele all’Adige, Italy; mmanica@fbk.eu (M.M.); roberto.rosa@unitn.it (R.R.); 4 Center for Health Emergencies, Bruno Kessler Foundation, 380123 Trento, Italy; 5 Center Agriculture Food Environment, University of Trento, 38098 San Michele all’Adige, Italy

**Keywords:** Culex quinquefasciatus, Anopheles gambiae complex, Anopheles arabiensis, Anopheles coluzzii, CDC-light traps, BG-sentinel traps

## Abstract

**Simple Summary:**

Mosquito-borne diseases such as malaria, arbovirosis and lymphatic filariasis are major public health issues, particularly in Africa. In order to predict the risk of transmission of these diseases and plan optimal mosquito control interventions, it is vital to have updated information of the mosquito species present, as each of them has a different capacity to transmit different pathogens, and to monitor how these species vary over time, also in relation to environmental and climatic changes. This is achieved by entomological monitoring carried out by various types of traps, whose collection efficacy may vary depending on the mosquito species and the ecological and climatic situation. We carried out collections in two villages in Senegal and showed evidence of a decline of malaria vector species and an increase of lymphatic filarial vectors. Moreover, we showed that using different traps to collect female mosquitoes may provide different estimates of mosquito species composition over time, depending on geographical setting and season. This is very relevant for a correct planning of mosquito monitoring and for appropriate interpretation of the results.

**Abstract:**

Mosquitoes-borne diseases are major public health issues particularly in Africa. Vector control interventions and human-made environmental/climatic changes significantly affect the distribution and abundance of vector species. We carried out an entomological survey targeting host-seeking mosquitos in two different ecological contexts—coastal and inland—in Senegal, by CDC-light and BG-sentinel traps. Results show high predominance of *Culex quinquefasciatus* (90%) and of *Anopheles arabiensis* within malaria vectors (46%), with mean numbers of females/trap/nights =8 and <1, respectively, reinforcing previous evidence of changes in species composition and abundance, highlighting thus increasing risk of transmission of filariasis and emerging arboviruses in the Senegambia region. From the methodological perspective, results show a higher specificity of BG traps for *Cx. quinquefasciatus* and of CDC traps for *An. gambiae* s.l. and highlight that, despite both traps target the host-seeking fraction of the population, they provide different patterns of species abundance, temporal dynamics and host-seeking activity, leading to possible misinterpretation of the species bionomics. This draws attention to the need of taking into account trapping performance, in order to provide realistic quantification of the number of mosquitoes per units of space and time, the crucial parameter for evaluating vector–human contact, and estimating risk of pathogen transmission.

## 1. Introduction

Mosquito-borne diseases such as malaria, arbovirosis and lymphatic filariasis are major public health issues particularly in Africa. Vector control interventions, as well as human made environmental and climatic changes, are significantly affecting the distribution and abundance of vector species [1]. In the Senegambia region, at the western extreme of west Africa, massive implementation of long-lasting insecticidal bed nets (LLINs) and indoor residual spraying (IRS) has been associated with: (i) a shift in malaria vector species [2,3]; (ii) a decline of mosquito biting rates [3]; (iii) a rapid evolution of insecticide resistance [4]; (iv) an increase of outdoor biting behavior of the local vectors [2]; (v) a high rate of hybridization between two main malaria vector species, *Anopheles coluzzii* and *An. gambiae* s.s. [5] possibly leading to a novel hybrid form resilient against introgression of medically-important loci and traits as hypothesized by Vicente et al. [6]. Moreover, the exponential increase of human population and a consequent increase in deforestation and rapid desertification as occurring in Senegal [7] are favoring species more adapted to polluted and arid environments, such as *Culex quinquefasciatus* among *Culicinae* [8], and *An. arabiensis* among the members of *An. gambiae* complex [9,10].

The careful monitoring of these major changes in mosquito vector populations is crucial to understand disease transmission and to plan/optimize anti-vector measures. Notably, the assessment and prediction of key entomological parameters (such as species abundance and dynamics, and host-seeking activity) relies on entomological monitoring which may be carried out by different trap devices.

The most widespread method to collect host-seeking mosquito females is the CDC-light trap, originally designed to collect *Anopheles* mosquitoes attracted by light [11,12] and later shown to be also effective for collecting *Culex* species [13]. A few studies highlighted that CDC-light trap is very efficient in collecting malaria vectors when located indoors close to a person sleeping under a net, while the performance of the trap outdoors is poorer [14,15]. Recently, CDC-light traps have been proposed as a reasonable alternative to human landing catches (HLC) for estimating *Anopheles* biting rates indoors [16].

Other traps have been developed in order to collect host-seeking females by releasing an artificial odor blend simulating that produced by a human host [17]. The BG-sentinel trap was initially designed to attract *Aedes albopictus* in temperate areas, as well as *Aedes aegypti* in tropical areas [18,19] and was later applied to collect host-seeking malaria vectors, as well as *Cx. quinquefasciatus* and other *Culicinae* species [20,21,22].

Few studies have been carried out to compare the performance of CDC and BG traps indoors and outdoors. In Burkina Faso, BG and CDC baited with the same chemical lure were compared, and CDC were shown to collect significantly higher numbers of *An. gambiae* s.l. females indoors, while the opposite occurred outdoors [22]. In Brazil, BG-malaria traps (BG with an upward airflow) were shown to be more efficient than CDC-traps in collecting *An. darlingi* outdoors [20].

We here present the results of an entomological survey carried out in two different ecological contexts—coastal and inland—in Senegal, and confirmed evidence of changes in species abundance and composition in the region. Results also highlight significant differences in the performance of CDC-light and BG-sentinel traps in collecting *Cx. quinquefasciatus* and *An. gambiae* s.l. (and its member species) females and in depicting their seasonal dynamics, raising concern on the use of entomological data to predict mosquito densities and evaluate vector–human contact necessary to feed epidemiological models and estimate the risk of pathogen transmission.

## 2. Materials and Methods

### 2.1. Study Areas

Mosquito collections were carried out in two sampling sites in Senegal about 300 km distant from each other. Madina Djikoye (13°38′59.55″ N, 16°19′36.52″ W; hereafter referred to as coastal village) is a rural village within the so call Peanut Basin, immediately north of the Gambian border and about 25 km from the coast. The ground is sandy, and the original wooded savannah has been almost entirely cleared by firewood, bushfires, drought or deforestation for agricultural activities. The climate is influenced by vicinity to the coast; highest temperatures are observed in April–May (40 °C) and October (35 °C), and lowest temperatures (<10 °C) in December–January. Two distinct seasons are present: a 7–8 month-long dry season (from November to May–June) and a 4–5 month-short rainy season (from June–July to October). The village is situated at 12 km distance from the field research station of Dielmo, which in 1990 was selected for a (still ongoing) longitudinal survey to investigate the determinants of malaria transmission, because of its high malaria prevalence [23,24]. Djinkore Mafing (13°42′23.35″ N, 13°39′14.9″ W; hereafter referred to as inland village) is located in Tambacounda region, in south-east Senegal, approximately 300 km from the coast. The region consists of land cover characterized by sandstone plateaus of the continental sedimentary basin with savannah woodlands, areas of agricultural parkland, and thin sections of gallery forest near river and bed streams. The region’s climate ranges between Sudan-Sahelian and Sudan-Guinean with an overall annual rainfall of 500 mm. The east of Senegal is one of the warmest regions of the country during the dry season. During the rainy season, the temperature decreases significantly due to land surface cooling associated with larger precipitation. The climate is characterized by two seasons: a 4–5 month-long rainy season (from May–June to October, with a peak in August–September, when rainfall can reach up to 200 mm) and a dry season, from November to May [10,25]. The main breeding sites around the villages are represented by rainwater puddles, well dugs, riversides, borrow pits. Septic tanks often not sealed are also present in both villages.

### 2.2. Mosquito Sampling and Processing

Host-seeking *Culicidae* collections were carried out during 3 samplings (September—hereafter, the rainy season, October—hereafter, the late rainy season, and November/December (2018)—hereafter, the dry season) for 4 consecutive nights each (from 6.00 p.m. until 7.00 a.m.) in 9 and 10 randomly selected houses in the coastal and inland village, respectively. During each sampling night, collections within each house either were carried out by CDC-light traps (John W. Hock Ltd., Gainesville, FL, USA; hereafter CDCs) or by BG-sentinel traps baited with BG lure (without CO_2_) (BioGents, Regensbourg, Germany; hereafter BGs), two traps of the same type were located in each house, one in a human sleeping room (indoor trap) and the second in the house courtyard (outdoor trap). In both cases, the traps were located close to a person sleeping under a bed-net. CDC and BG traps were rotated daily among houses in order to avoid sampling biases. Ethical approval for the study was granted by Ministère de la Santé et de l’Action Sociale (Comité National d’Ethique pour la Recherche en Santé, N0000049 MSA/DPRS/CNERS, Dakar, Senegal, 27 July 2018).

*Anophelinae* and *Culicinae* mosquitoes were identified at species level using morphological taxonomic keys [26]. *Anophelinae* samples were labeled and stored for molecular analyses in Eppendorf tubes containing silica gel desiccant.

Genomic DNA of *An. gambiae* complex specimens was extracted from single mosquitoes (legs or head + thorax) following [27]. Extracted DNA was then used as template for the PCR-based species identification [28].

### 2.3. Statistical Methods

Species diversity was calculated for each trap type in the two villages by Simpson’s Index:

(1) $$D\;=\frac{\sum_{i=1}^{S}ni\;\left(ni-1\right)}{N\;\left(N-1\right)}\;$$

where *S* = number of *Culicinae* or *Anophelinae* in the area, ni = number of individuals in species-i and *n* = total number of *Culicinae* or *Anophelinae* in the area. The Simpson’s index gives the probability without replacement that two individuals taken at random from a sample are of the same species. It ranges between 0 (greater diversity) and 1 (lower diversity).

To evaluate the performance of the two trap types, we assessed by regression analysis the relationship between abundance of either *Cx. quinquefasciatus* or *An. gambiae* s.l. females and the following covariates: trap type (CDC and BG traps), trapping location (indoor vs outdoor), and month of collections (from rainy to dry season). We used the number of *Cx. quinquefasciatus* or *An. gambiae* s.l./trap/night as response variable and assumed that it followed a negative binomial distribution (using a Poisson distribution resulted in overdispersion). Initially, we included in the full model the three covariates and all their interactions to check whether the sampling is influenced by trapping location and months of collections. We tested both generalized linear models (GLM-1) and generalized linear mixed effect models (GLMMs-1) approach by considering houses as random effect. We decided whether to include or not the random effect by comparing the models by the Akaike information criteria (AIC). Then, after selecting the appropriate random structure we performed variable selections by fitting all possible sub-models and ranking them by AICc. Finally, we discuss in the result section, the best parsimonious model among the subset of models having delta AICc < 4. The model was fitted for each village separately.

The above described model without random effects was also used to estimate the mean number of mosquitoes/person/night in each village (assuming that this corresponds to the number of collected mosquitoes/trap/night [16]) considering only trap type as covariate (GLM-2).

For *An. gambiae* s.l., we computed the relative frequencies of *An. arabiensis*, *An. gambiae* s.s., *An. coluzzii,* hybrids and *An. melas* in each village and in each month of collection. We analyzed the performance of trap type, in terms of the probability of detection of *An. arabiensis, An. gambiae* s.s. and *An. coluzzii* females, as a function of period of collections and trapping location, assuming that the response variable (i.e., presence/absence of mosquitoes/trap/night) followed a Bernoulli distribution. We tested both generalized linear models (GLM-3) and generalized linear mixed models (GLMM-3) approach by considering house as a random effect. The same selection procedure described above was applied to select and discuss the best parsimonious model.

We used R statistical software version 3.6.3 [29] and lme4,MuMIn, MASS packages for all statistical analysis. To avoid convergence problems, the maximum number of iterations of the glmer. nb function in the lme4 package was increased to 10,000.

## 3. Results

### 3.1. Species Composition and Descriptive Statistics

**Figure 1:**
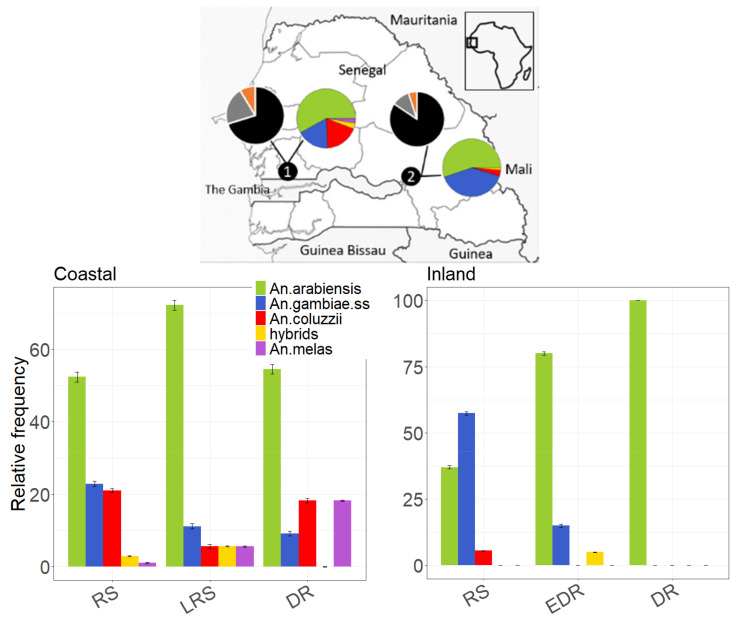
Relative frequency of *Culicidae* species in a coastal and in an inland village of Senegal (pies) and of Anopheline species in the four collection periods (histograms). **Upper** panel: *Culex quinquefasciatus* (*n* = 2899; black), Other *Culicinae* species (*n* = 624; grey), Anopheline (*n* = 266; orange). *Anopheles arabiensis* (green) *Anopheles coluzzii* (red), *An. gambiae* (blue) their hybrids (yellow) and *An. melas* (purple). Coastal village: RS (Rainy Season; 1–4 September 2018), LRS (Late Rainy Season; 10–13 October 2018), DS (Dry Season; 29–30 November–1–2 December 2018). Inland village: RS (Rainy Season; 27–30 September 2018), EDS (Early Dry Season; 4–7 November 2018), DS (Dry Season; 17–20 November 2018).

A total of 4192 (3792 females and 400 males) *Culicidae* were collected by CDC (1557 females, 338 males) and BG (2235 females; 62 males) traps in 24 sampling nights carried out in the coastal and inland village from rainy to dry season 2018 (Tables S1 and S2). Overall, *Culicinae* largely prevails (90%) over *Anophelinae* in both villages (Figure 1, left panel).

As expected in case of nocturnal collections, *Culicinae* are represented by *Culex* (87% and 95% in the coastal and inland, respectively), *Aedes* (7% and 5%) and *Mansonia* (5% and 0.1%). Highest *Culicinae* species diversity is observed in BG collections (coastal village: D-BG = 0.18, D-CDC = 0.69; inland village: D-BG = 0.17, D-CDC = 0.39). *Cx. quinquefasciatus* is the most abundant *Culicinae* species in both villages (80% and 88% in the coast and inland, respectively). Other species found at frequencies >1% are *Culex nebulosus* (6%), *Cx policilipes* (1.4%), *Ae vexans* (5%) and *Mansonia uniformis* (4%) in the coastal village, and *Ae vexans* (4%), *Cx tritaeniorhynchus* (1%) and *Ae aegypti* (1.5%) and *Cx tigripes* (1%) in the inland village (Tables S1 and S2).

A sharp temporal dynamic of *Cx. quinquefasciatus* is observed in the inland village where 9% of the specimens were collected in the rainy season and 53% in the dry season, but not in the coastal one (28% in the rainy season and 37% in the dry season).

*Anopheles gambiae* s.l. represents 83% and 96% of the total *Anophelinae* captured in the coastal and inland village, respectively. Few individuals of *An. funestus* and other less public health relevant anopheline species (i.e., *An. ziemanni, An. rufipes, An. domicola*, *An. nili*) are also found. As opposed to *Culicinae*, the highest *Anopheline* species diversity is observed in CDC-collections (coastal village: D-BG = 0.64, D-CDC = 0.42; inland village: D-BG = 0.25, D-CDC = 0.19). Among the 207 *An. gambiae* s.l. females successfully genotyped out of the 214 collected (Table S3; Figure 1), *An. arabiensis* represents 55% and 52% of the coastal and inland collected samples, respectively. *Anopheles coluzzii* and *An. gambiae* are both found at frequencies around 20% in the coastal village, while the latter species largely prevails (42%) in the inland one. *Anopheles gambiae*/*coluzzii* hybrids represent 3% and 1% of the total samples collected in the coastal and inland village, respectively. *Anopheles melas* is found only in the coast (3%), as expected due to its salt-water habitat preferences. Relative frequencies of *An. gambiae* s.l. species in the four collection periods are shown in Figure 1 (right panel).

Given the strong prevalence of *Cx. quinquefasciatus* among *Culicinae* and *An. gambiae* s.l. among *Anophelinae*, as well as their relevant role as vectors of filarial worms and malaria in Senegal respectively, comparisons of trap performances were carried out for these species only.

### 3.2. Performance of CDC and BG Traps in Collecting Culex quinquefasciatus Females as a Function of Month of Collection and Trapping Location

BG traps collected 57% and 86% of the overall *Cx. quinquefasciatus* sample in the coastal (*n* = 1493) and inland village (*n* = 1406), respectively. Within the BG-trap overall sample, 57% and 77% of the females were collected outdoors, respectively, while the opposite trend is observed for CDC-traps (44% and 47%, respectively).

Results of model selection show lower AIC for GLMM-1 (coastal village: AIC = 1186, inland village AIC = 1119) than for GLM-1 (coastal village AIC = 1194, inland village = 1128), indicating the need to include house of collections as random effect.

**Figure 2:**
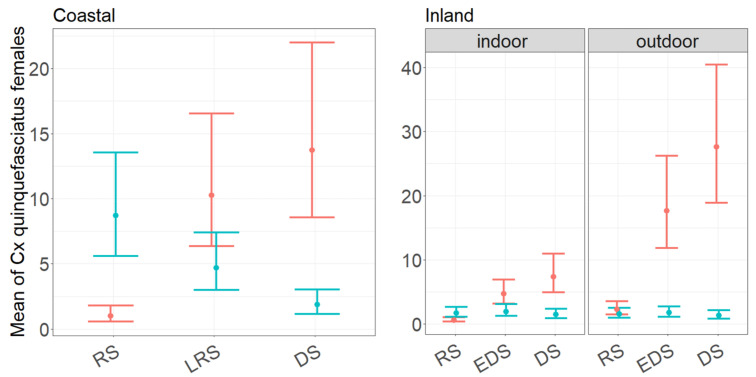
Mean estimated abundance of *Culex quinquefasciatus* host-seeking females/night in a coastal (**Left**) and inland (**Right**) village in Senegal in 2018, as predicted by GLMM-1 model. Red and blue lines = predicted regression lines from models fit on data collected in BG and CDC, dots = mean estimated abundance of *Cx. quinquefasciatus* females. Vertical lines = 95% confidence intervals. Coastal village: RS (Rainy Season; 1–4 September 2018), LRS (Late Rainy Season; 10–13 October 2018), DS (Dry Season; 29–30 November–1–2 December 2018). Inland village: RS (Rainy Season; 27–30 September 2018), EDS (Early Dry Season; 4–7 November 2018), DS (Dry Season; 17–20 November 2018).

In the coastal village, the covariate selection indicates that the mean abundance of *Cx. quinquefasciatus* females correlates with trap type, period of collection and with the interactions between trap type and period of collection, but not with the indoor/outdoor location of the traps (Table S4). The interaction between trap type and period of collections is statistically significant, indicating that CDC and BG traps depict a different temporal pattern, i.e., the mean predicted abundance per night decreases from rainy to dry season when estimated based on CDC collections, but increases when estimated based on BG ones (Figure 2). Overall, the BG performance is higher for all periods of collection (except in rainy season) compared to CDC.

In the inland village, the mean abundance of *Cx. quinquefasciatus* host-seeking females correlates with all variables and with the interactions between trap type and period of collection and between trap type and trapping location (Table S5). The interaction between trap type and period of collection is statistically significant and follows the same patterns observed in the coastal village (Figure 2). Moreover, the interaction between trap type and trapping location is statistically significant, i.e., higher values are predicted for BGs located outdoors vs indoors, while the opposite is observed for CDC. A summary of GLMM-1 models results in both village was reported in Table S6.

### 3.3. Performance of CDC and BG Traps in Collecting Anopheles gambiae s.l. Females as Function of Month of Collection and Trapping Location

CDC traps collected 96% of the overall *An. gambiae* s.l. sample in the coastal village (*n* = 150) and 79% in the inland one (*n* = 82). Within CDC coastal and inland village samples, 59% and 81% of the females were collected indoors, respectively, while BG collections show an opposite trend (34% and 42%, respectively).

Results of the model selection applied to GLM-1 and GLMM-1, developed to test the performance of the two trap types to collect *An. gambiae* s.l. host-seeking females as function of period of collection and trap type, show no effect in including houses of collections as random effect (coastal village: AIC GLM-1 = 397, AIC GLMM-1 = 398; inland village AIC GLM-1 = 301, AIC GLMM-1 = 303). 

**Figure 3:**
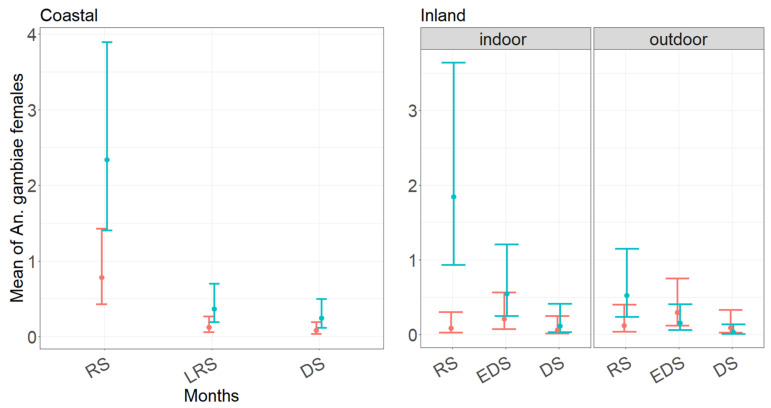
Mean estimated abundance of *Anopheles gambiae* s.l. host-seeking females/night in a coastal (**Left**) and inland (**Right**) village in Senegal in 2018, as predicted by GLMM-1 model. Dots = mean of *An. gambiae* s.l. females. Red and blue lines = predicted regression lines from models fit on data collected in BG and CDC, respectively. Lines = 95% confidence intervals. Coastal village: RS (Rainy Season; 1–4 September 2018), LRS (Late Rainy Season; 10–13 October 2018), DS (Dry Season; 29–30 November–1–2 December 2018). Inland village: RS (Rainy Season; 27–30 September 2018), EDS (Early Dry Season; 4–7 November 2018), DS (Dry Season; 17–20 November 2018).

In the coastal village, the covariate selection indicates that the mean abundance of *An. gambiae* s.l. host-seeking females per night correlates with trap type and period of collection, but not with the indoor/outdoor location of the traps (GLM-1, Table S7). No interaction among variables is observed. Mean abundance of *An. gambiae* s.l. is significantly higher in CDC-collections (Figure 3) and in rainy season, independently from the trap type (Figure 3).

In the inland village, covariate selection indicates that the mean *An. gambiae* s.l. abundance correlates with all variables and includes the interaction between trap type and period of collection, as well as trap type and trapping location (Table S8). The significance of the interaction of trap type and period of collection highlights a peak of abundance in rainy season and a decrease afterwards in the case of CDC collections, while no statistical difference is observed among months of collection for BGs (*p*-value in early dry season = 0.19; *p*-value in dry season = 0.69; Table S8; Figure 3). In addition, CDC depicts higher abundances indoors rather than outdoors during the whole sampling period (*p*-value = 0.027), while no statistical difference is observed between indoor and outdoor BG collections (*p*-value = 0.52) (Table S8). A summary of GLM-1 models results in both village was reported in Table S9.

### 3.4. Performance of CDC and BG in Assessing the Probability to Detect Members of Anopheles gambiae Complex

Due to low number of *An. gambiae* s.l. collected despite the relevant sampling effort, we modelled the probability of detection of *An. arabiensis* (AR), *An. gambiae* (GA) and *An. coluzzii* (CO) (the latter one only in coastal village, due to low sample size in inland one) host-seeking females in each village, rather than their mean abundance. Results of the model selection show a lower AIC for GLM-3 (coastal village: AR = 208, GA = 108, CO = 113; inland village: AR = 165, S = 100) than for GLMM-3 (coastal village AR = 210, GA = 110, CO = 111; inland village: AR = 166, CO = 102), indicating no need to include house of collections as random effect.

**Figure 4:**
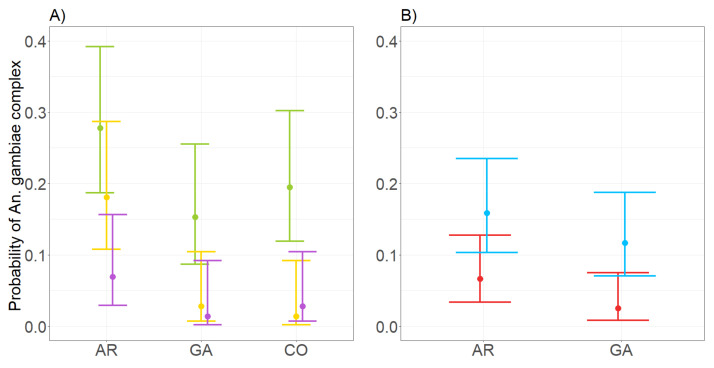
Probability to collect *Anopheles gambiae* complex species in coastal (**A**) and inland (**B**) village in Senegal as modelled by GLM-3. Left panel (**A**): green (September), yellow (October), and purple (November–December) dots = probability to collect *Anopheles gambiae* complex species. Right panel (**B**): blue dots = probability to collect *An. arabiensis* and *An. gambiae* with CDC-light. Red dots = probability to collect *An. arabiensis* and *An. gambiae* with BG-sentinel traps. Lines = 95% confidence intervals. AR = *An. arabiensis*, GA = *An. gambiae s.s*, CO = *An. coluzzii*.

In the coastal village, results of covariate selection procedure indicate that the only statistically significant covariate to explain the probability to find a specimen is the month of collection (Table S10). A reduction of the predicted probability to collect the three species is observed from the first to the last sampling period (Figure 4).

In the inland village, results of covariate selection procedure indicate that the probability to find a specimen depends instead on trap type only (Table S11), i.e., a higher probability to collect *An. arabiensis* and *An. gambiae* with CDC than with BG traps. The odds to collect *An. arabiensis* and *An. gambiae* through CDC traps is 2.6 (95% CI 1.13–6.63) and 5.1 (95% CI: 1.63–22.78) times higher compared to BG traps, respectively (Figure 4).

## 4. Discussion

### 4.1. Performance of CDC and BG Traps in Collecting Culex quinquefasciatus Host-Seeking Females

BG-traps were found to trap larger *Culicinae* species diversity than CDC-traps and to be significantly more performant in collecting the most abundant species, *Cx. quinquefasciatus,* (except in the collection carried out in the coastal village in rainy season, when the species abundance was lowest). A slightly higher species diversity indices for BG trap was also reported in a comparison among four trapping devices (including BG and CDC) carried out in Germany [30]. Consistent results on relative trap performance were found in a study comparing BG-traps baited with octenol and CO_2_ with CDC-traps baited with CO_2_ in a 3 × 3 Latin-square experiment in the Samoan Islands [31]. An opposite result was instead obtained when using our same approach in China [32], suggesting that trap relative performance may be affected by eco-climatic conditions and/or by genetic traits of the target populations, as well as use of different attractants.

When exploiting the data to predict the number of collected females in relation to different variables, the trap type variable was always retained in the statistical models by the variable selection procedure and always interacted with the rest of covariates. The statistically significant interaction effect between trap type and month of collection reflects an opposite temporal dynamic depending on type of trap used for the sampling. The significant interaction between trap type and trap location observed in the inland village indicates that the two traps depict opposite host-seeking patterns, (i.e., a preference for outdoor and indoor biting depicted by BG and CDC traps, respectively), suggesting that the capture rate of the two trap types is affected by the indoor vs. outdoor location.

To our knowledge, this represents the first study addressing the performance over time of CDC and BG traps to depict *Cx. quinquefasciatus* temporal dynamics and host-seeking behavior. If the differences highlighted with reference to season, as well as to the indoor/outdoor location of the traps will be confirmed in other settings, this will need to be taken into careful consideration when trap data are exploited to plan vector control interventions and to predict absolute species abundance or risk of disease transmission.

### 4.2. Performance of CDC and BG Traps in Collecting Species of the Anopheles gambiae Complex

CDC traps are shown to be the preferable trap to samples *Anopheline* species compared to BG-trap. They yielded a higher species diversity and were significantly more performant in collecting *An. gambiae* s.l. females both indoors and outdoors in both villages. CDC also showed to have a higher probability than BGs in detecting *An. gambiae* s.s. and *An. arabiensis* (in the inland village). In the only study comparing the performance of the two traps (both baited with the same lure), CDC traps consistently collected more *An. coluzzii* females than BGs when traps were located indoors, but BG collections massively outperform CDC ones in the outdoor environment, leading the authors to strongly recommend BG traps for outdoor sampling [22]. This recommendation is weakened by present results, which may suggest that, as in the case of *Cx. quinquefasciatus,* the relative performance of the two traps may be affected by eco-climatic conditions and/or by genetic traits of the target populations. However, it should be noted that in Pombi et al. [22] both traps were baited with lure (used in the present study only in BG traps) and with a source of CO_2_ produced by sugar-fermenting yeast (not used in present study).

### 4.3. Culex quinquefasciatus Female Abundance, Seasonality, and Indoor/Outdoor Preferences in the Coastal and Inland Village

Our experimental design (i.e., traps located close to a single person sleeping under the bed net either indoors or outdoors) was aimed to allow to approximate the number of females/trap/night to the number of females/person/night (as suggested by [16,22]). Under this assumption, the average of *Cx. quinquefasciatus* females/person/night in the study period (based on BG collections which performed better than CDC for this species, see above) can be estimated to range between 8.7 (95% CI 6.5–11.8) and 5.5 (95% CI 4.2–7.2) in the coastal village and between 10 (95% CI 7.9–12.7) and 1.7 (95% CI 1.3–2.3) in the inland village (see M&M Statistic Method—GLM-2; Figure S1, Tables S12 and S13). If we consider the mean negative binomial distribution, we can estimate that in the sampling period of highest *Cx. quinquefasciatus* abundance, the females/person/night in 5% of human host population can reach up to 44 and 34 in coastal and inland village, respectively. These estimates of high mosquito–human contact are likely associated to presence of in anthropogenic polluted waters rich in organic matter in both study villages (e.g., septic tanks), and suggest high risk of disease transmission with particular reference to lymphatic filariasis, which is endemic in Senegal [33]. Results from the inland village are consistent with recent report of high *Cx. quinquefasciatus* abundance from south-eastern Senegal [8].

Results do not highlight any clear indoor/outdoor preference in *Cx. quinquefasciatus* host-seeking behavior, i.e., the estimated species abundance is higher outdoors based on BG trap collection, and indoors based on CDC traps. Overall, this suggests that in our sampling sites *Cx. quinquefasciatus* does not show the strong endophagic behavior reported from other West African sites [8,34]. It is, however, to be reminded that the species is known to exhibit different biting habits worldwide [34], indicating a high behavioral plasticity. It is possible to hypothesize that the lack of clear endophagy observed in this study could be due to species adaptation to the increased number of people protected by LIINs and/or IRS in the indoor environment in the frame of national malaria control plans.

### 4.4. Anopheles gambiae Complex Species Abundance, Seasonality, and Indoor/Outdoor References in the Coastal and Inland Village

The average number of *An. gambiae* s.l. females/trap/night (i.e., females/person/night) in the coastal village is shown to be constantly < 1 (Figure S2, Tables S14 and S15). This value is consistent with the significant reduction in *An. gambiae* s.l. biting rate observed from 2006 to 2016, following a large scale campaign of LLINs distribution in the coastal region [3,35]. Our results suggest a possible even greater decrease, as estimates are based on trap collections rather than HLCs, which are known to be more efficient in collecting human biting Anophelines [36,37]. No previous data are available from the inland village/region, where numbers of *An. gambiae* s.l. females/trap/night are even lower than in the coast (0.55, 95% CI 0.34/0.90) (Figure S2). However, the lower average of mosquitoes/traps/night observed in the inland compared to the coastal village is not in agreement with the higher malaria incidence reported in south-eastern regions of Senegal [38] and Gambia [39]. The temporal dynamic of *An. gambiae* s.l. in the two sampling villages (as depicted by CDC collections) is consistent with previous data showing a peak of abundance in rainy season and a sharp decreases towards the end of the rainy season and the beginning of dry season [3].

*Anopheles arabiensis* prevails in both villages over the other members of the complex (except that in the inland village at the beginning of the survey). The high prevalence of the species in the coastal village is consistent with recent observations from the nearby village of Dielmo and neighboring coastal villages [2,35]. Furthermore, our results confirm a range expansion for *An. arabiensis*, from north to south, likely as a result of increasing drought and/or human activities (such as deforestation and urbanization), as suggested by [10].

*Anopheles coluzzii* and *An. gambiae* are found with similar frequencies in the coastal village—where both rain-dependent and semi-permanent breeding sites associated to small permanent rivers (Djikoye River and Nema River) are present—while *An. gambiae* predominates in the inland one, where only rain-dependent breeding sites are present. These observation are in agreement with previous reports from coastal [40] and south-eastern Senegal [10,25,40]. The report of *An. coluzzii* and *An. gambiae* hybrids at frequencies of 3% and 1% in the coastal and inland village, respectively, reflects a level of hybridization higher than in the rest of the species’ sympatric range in West-Africa [41], but overall in the range of other observations from Senegal and neighboring Gambia [10,39,40]. However, more in the details, hybrid frequency reported from the coastal village appears to be lower than in previous reports from coastal Senegalese region [40], but higher than those reported inland regions [10], suggesting that the breakdown in the reproductive isolation between the two species may be not restricted to the coastal region and/or expanding inland.

Results do not highlight strong preference of the three *An. gambiae* complex members for indoor vs outdoor host-seeking in both coastal and inland village, as trap location does not explain the variability of the data in the models. Lack of endophagic/exophagic preference was already observed in *An. arabiensis* in Dielmo and in other areas of eastern Senegal [27].

## 5. Conclusions

Our study is focused on mosquito species most relevant from the public health perspective due their closely association to humans, as it reports results from collections carried out inside or close to human habitations. Results reinforce previous evidence of an overall decline of malaria vector species in coastal and inland southern Senegal and of a parallel increase in *Cx. quinquefasciatus* abundance, highlighting risk of transmission of endemic pathogens, such as *Wuchereria bancrofti*, and emerging pathogens such as Rift Valley Fever and West Nile in the country. This reflects an opposite effect of selective pressures of human-made origin (e.g., decreased presence of unpolluted breeding sites due to urbanization and desertification) on different vector species. Results also confirm predominance of *An. arabiensis* over other members of the *An. gambiae* complex not only in the coastal area, where this was already reported, but also inland, likely as a result of increasing drought and anthropogenic environmental changes, including extensive LLIN and IRS exploitation.

From the methodological perspective, results highlight a higher specificity of BG traps for *Cx. quinquefasciatus* and of CDC traps for Anopheline vectors. Moreover, results also unexpectedly showed that the relative performance of two traps varies in relation to the month of collection and to the trap indoor/outdoor location, despite both traps target the host-seeking fraction of the population. This implies that predictions of the mean numbers of mosquito/person/night, as well as the of the species temporal dynamic and host-seeking behavior, may vary depending on the trapping device used, thus questioning the use of entomological data to feed epidemiological models or to plan and assess the results of control interventions. Future works should address this weakness in malaria vector monitoring, for example by applying mathematical/statistical models able to account for different trapping performance in order to provide realistic quantification of mosquito density (i.e., number of mosquitoes in units of space and time), the crucial parameter for the evaluation of vector–human contact and for the development of epidemiological models to estimate the risk of pathogen transmission.

## Data Availability

The data are available online at https://github.com/Chia1992/Senegal_2020.

## Supplementary Materials

The following are available online at https://www.mdpi.com/article/10.3390/insects12080692/s1, Table S1, Culicidae females collected in a coastal and in an inland village in Senegal by CDC-light and BG-sentinel traps (n = 20 trap-night for both traps) located indoors and outdoors from September to November 2018; Table S2, Culicidae males collected in a coastal and in an inland village in Senegal by CDC-light and BG-sentinel traps (n = 20 trap-night for both traps) located indoors and outdoors from September to November 2018; Table S3, Females of Anopheles gambiae s.l. species collected indoors and outdoors in a coastal and an inland village in Senegal by CDC-light and BG-sentinel traps from September to November 2018; Table S4, Result of best parsimonious of GLMM-1 of Culex quinquefasciatus female abundance in a coastal village in Senegal; Table S5, Result of the best parsimonious of GLMM-1 of Culex quinquefasciatus female abundance in an inland village in Senegal; Table S6, Summary of the mean of Culex quinquefasciatus in the coastal and inland village predicted by GLMM-1; Table S7, Result of best parsimonious of GLM-1 model of Anopheles gambiae s.l. female abundance in the coastal village; Table S8, Result of the best parsimonious of GLM-1 of Anopheles gambiae s.l. female abundance in an inland village in Senegal; Table S9, Summary of the mean of Anopheles gambiae s.l. in a coastal and inland village in Senegal predicted by GLM-1; Table S10, Result of the best parsimonious of GLM-3 of Anopheles arabiensis, An. gambiae and An. coluzzii probability in a coastal village in Senegal; Table S11, Result of the best parsimonious of GLM-3 of Anopheles arabiensis and An. gambiae probability in a inland village in Senegal; Table S12, Result of GLM-2 of the mean of Culex quinquefasciatus/trap/night in a coastal village in Senegal; Table S13, Result of GLM-2 of the mean of Culex quinquefasciatus/trap/night in an inland village in Senegal; Table S14, Result of GLM-2 of mean Anopheles gambiae s.l. females/trap/night in a coastal village in Senegal; Table S15, Result of GLM-2 of mean Anopheles gambiae s.l. females/trap/night in an inland village in Senegal; Figure S1, Average number of Culex quinquefasciatus host-seeking females/trap/night collected by BG-sentinel and CDC-light traps in a coastal (Left) and in an inland (Right) village in Senegal as estimated by GLM-2. Dots=average number mosquito females/person/night. Black vertical lines=95% confidence intervals; Figure S2, Average number of Anopheles gambiae s.l. host-seeking females/trap/night collected by BG-sentinel and CDC-light traps in a coastal (Left) and in an inland (Right) village in Senegal, as estimated by GLM-1. Dots=average number mosquito females/trap/night. Vertical lines=95% confidence intervals.
