# Supplementary material for: Entomological Survey Confirms Changes in Mosquito Composition and Abundance in Senegal and Reveals Discrepancies among Results by Different Host-Seeking Female Traps
Source: Insects. 2021 Jul 31;12(8):692. doi: 10.3390/insects12080692 (PMC8396443; doi:10.3390/insects12080692)
Supplement: Supplementary file 1 [file insects-12-00692-s001.zip › insects-1285400-supplementary.pdf]

Table S1. Culicidae females collected in a coastal and in an inland village in Senegal by CDC-light and BG-sentinel traps (*n*= 20 trap-night for both traps) located indoors and outdoors from September to November 2018.

Species	Coastal												Inland											
	September				October				Late-November				September				Early November				Mid-November			
	Indoor		Outdoor		Indoor		Outdoor		Indoor		Outdoor		Indoor		Outdoor		Indoor		Outdoor		Indoor		Outdoor	
	**CDC**	**BG**	**CDC**	**BG**	**CDC**	**BG**	**CDC**	**BG**	**CDC**	**BG**	**CDC**	**BG**	**CDC**	**BG**	**CDC**	**BG**	**CDC**	**BG**	**CDC**	**BG**	**CDC**	**BG**	**CDC**	**BG**
Culex quinquefasciatus	243	21	147	20	91	169	92	177	36	163	44	290	54	11	18	54	30	118	47	315	27	135	32	565
Culex nebulosus	65	5	61	2	7	0	12	0	0	4	0	2	0	0	0	0	0	0	0	0	0	0	0	0
Culex poicilipes	3	0	3	0	7	0	3	0	0	2	6	8	0	0	0	0	1	0	0	2	0	0	0	0
Culex tritaeniorhynchus	0	0	0	0	2	0	0	0	0	0	0	0	0	0	0	0	0	3	0	3	2	0	1	7
Culex tigripes	0	0	0	0	0	0	0	0	0	0	0	0	2	0	0	3	0	0	0	2	0	0	0	9
Culex ethiopicus	0	0	0	0	0	0	0	0	0	0	0	0	0	0	0	0	3	0	2	0	0	0	0	0
Mansonia africana	5	0	1	0	0	0	0	0	0	0	4	0	0	0	0	0	0	0	0	0	0	0	0	0
Mansonia uniformis	29	0	10	0	32	0	13	0	5	0	11	1	0	0	0	0	0	0	0	0	0	0	0	0
Aedes aegypti	4	0	10	0	4	0	0	0	0	0	0	0	6	0	6	5	0	0	0	0	0	0	1	1
Aedes ochraceus	0	0	0	0	1	0	0	0	2	0	3	0	2	1	2	7	0	0	0	1	0	0	0	0
Aedes vexans	0	0	0	0	32	6	57	26	0	0	0	0	13	5	22	64	0	0	0	0	0	0	0	0
**Total of Culicinae**	**349**	**26**	**232**	**22**	**176**	**175**	**177**	**203**	**43**	**169**	**68**	**301**	**77**	**17**	**48**	**133**	**34**	**121**	**49**	**323**	**29**	**135**	**34**	**582**
Anopheles gambiae s.l.	73	1	40	1	10	0	12	1	2	1	7	2	45	3	6	1	5	4	6	6	3	0	0	3
Anopheles funestus	0	0	1	0	5	0	0	0	0	0	0	0	0	0	0	0	0	0	0	0	0	0	0	0
Anopheles ziemanni	0	0	7	0	4	0	4	1	0	0	1	0	0	0	0	0	0	0	0	0	0	0	0	0
Anopheles rufipes	0	0	3	0	1	0	2	1	0	0	0	3	1	0	0	0	1	0	0	0	0	0	0	0
Anopheles domicola	1	0	0	0	0	0	0	0	0	0	0	0	0	0	0	0	0	0	0	0	0	0	0	0
Anopheles nili	0	0	0	0	0	0	1	0	0	0	0	0	0	0	0	0	0	0	0	0	0	0	0	0
**Total Anopheline**	**74**	**1**	**51**	**1**	**20**	**0**	**19**	**3**	**2**	**1**	**8**	**5**	**46**	**3**	**6**	**1**	**6**	**4**	**6**	**6**	**3**	**0**	**0**	**3**

Table S2. Culicidae males collected in a coastal and in an inland village in Senegal by CDC-light and BG-sentinel traps (*n*= 20 trap-night for both traps) located indoors and outdoors from September to November 2018.

Species	Coastal												Inland											
	September				October				Late-November				September				Early November				Mid-November			
	Indoor		Outdoor		Indoor		Outdoor		Indoor		Outdoor		Indoor		Outdoor		Indoor		Outdoor		Indoor		Outdoor	
	**CDC**	**BG**	**CDC**	**BG**	**CDC**	**BG**	**CDC**	**BG**	**CDC**	**BG**	**CDC**	**BG**	**CDC**	**BG**	**CDC**	**BG**	**CDC**	**BG**	**CDC**	**BG**	**CDC**	**BG**	**CDC**	**BG**
Culex quinquefasciatus	161	14	100	12	13	0	17	11	4	0	6	2	0	1	0	1	0	1	0	0	0	0	0	0
Culex nebulosus	0	0	0	0	0	0	0	0	0	0	0	0	0	0	0	0	0	0	0	0	0	0	0	0
Culex poicilipes	0	0	0	0	0	0	0	0	0	0	2	0	0	0	0	0	0	0	1	0	0	0	0	3
Culex tritaeniorhynchus	0	0	0	0	0	0	0	0	0	0	0	0	0	0	0	0	0	0	0	0	0	0	0	0
Culex tigripes	0	0	0	0	0	0	0	0	0	0	0	0	0	0	0	0	0	0	0	0	0	0	0	0
Culex ethiopicus	0	0	0	0	0	0	0	0	0	0	0	0	0	0	0	0	0	0	0	0	0	0	0	0
Mansonia africana	0	0	1	0	0	0	0	0	0	0	0	0	0	0	0	0	0	0	0	0	0	0	0	0
Mansonia uniformis	0	0	0	0	0	0	0	0	0	0	0	0	0	0	0	0	0	0	0	0	0	0	0	0
Aedes aegypti	0	1	0	0	1	0	0	0	0	0	2	0	1	0	0	0	0	1	1	0	1	1	0	0
Aedes ochraceus	0	0	0	0	0	0	0	0	0	0	0	0	0	0	0	0	0	0	0	0	0	0	0	0
Aedes vexans	0	0	0	0	0	0	0	0	0	0	0	0	0	0	0	0	0	0	0	0	0	0	0	0
**Total of Culicinae**	**161**	**15**	**101**	**12**	**14**	**0**	**17**	**11**	**4**	**0**	**10**	**2**	**1**	**1**	**0**	**1**	**0**	**2**	**2**	**0**	**1**	**1**	**0**	**3**
Anopheles gambiae s.l.	8	0	11	3	0	3	2	0	0	1	0	4	3	0	2	2	0	0	1	1	0	0	0	0
Anopheles funestus	0	0	0	0	0	0	0	0	0	0	0	0	0	0	0	0	0	0	0	0	0	0	0	0
Anopheles ziemanni	0	0	0	0	0	0	0	0	0	0	0	0	0	0	0	0	0	0	0	0	0	0	0	0
Anopheles rufipes	0	0	0	0	0	0	0	0	0	0	0	0	0	0	0	0	0	0	0	0	0	0	0	0
Anopheles domicola	0	0	0	0	0	0	0	0	0	0	0	0	0	0	0	0	0	0	0	0	0	0	0	0
Anopheles nili	0	0	0	0	0	0	0	0	0	0	0	0	0	0	0	0	0	0	0	0	0	0	0	0
**Total Anopheline**	**8**	**0**	**11**	**3**	**0**	**3**	**2**	**0**	**0**	**1**	**0**	**4**	**3**	**0**	**2**	**2**	**0**	**0**	**1**	**1**	**0**	**0**	**0**	**0**

Table S3. Females of *Anopheles gambiae* s.l. species collected indoors and outdoors in a coastal and an inland village in Senegal by CDC-light and BG-sentinel traps from September to November 2018.

**Species**	**Coastal village**												**Total coastal village**
	September (1–5)				October (10–14)				Nov (29–30)–Dec (1–3)				
	Indoors		Outdoors		Indoors		Outdoors		Indoors		Outdoors		
	CDC	BG	CDC	BG	CDC	BG	CDC	BG	CDC	BG	CDC	BG	
Anopheles arabiensis	36	6	7	6	6	1	4	2	1	1	3	1	74
Anopheles coluzzii	7	3	10	2	0	0	1	0	0	0	1	1	25
Anopheles gambiae	11	3	6	4	1	0	1	0	0	0	1	0	27
Anopheles gambaie/coluzzii hybrid	2	0	1	0	0	0	1	0	0	0	0	0	4
Anopheles melas	0	0	1	0	0	0	1	0	0	0	2	0	4
**Total**	**56**	**12**	**25**	**12**	**7**	**1**	**8**	**2**	**1**	**1**	**7**	**2**	**134**
	**Inland village**												**Total inland village**
	September (27–30)–Oct (1)				November (4–8)				November (17–21)				
	Indoors		Outdoors		Indoors		Outdoors		Indoors		Outdoors		
	CDC	BG	CDC	BG	CDC	BG	CDC	BG	CDC	BG	CDC	BG	
Anopheles arabiensis	18	0	1	1	4	2	6	4	3	0	0	3	42
Anopheles coluzzii	3	0	0	0	0	0	0	0	0	0	0	0	3
Anopheles gambiae	24	3	4	0	1	0	0	2	0	0	0	0	34
Anopheles gambaie/coluzzii hybrid	0	0	0	0	0	1	0	0	0	0	0	0	1
Anopheles melas	0	0	0	0	0	0	0	0	0	0	0	0	0
**Total**	**45**	**3**	**5**	**1**	**5**	**3**	**6**	**6**	**3**	**0**	**0**	**3**	**80**

Table S4. Result of best parsimonious of GLMM-1 of *Culex quinquefasciatus* female abundance in a coastal village in Senegal. September and BG-sentinel trap taken as reference levels. For the coastal village, number of observations (trap/house/night) = 215. Standard deviation of random effects = 0.36.

Variable	Coeff.	SE	z-value	$Pr(>\lvert z \rvert)$
**(Intercept)**	0.009	0.294	0.035	0.972
**October**	2.316	0.34	6.792	<0.0001
**Nov-Dec**	2.607	0.341	7.619	<0.0001
**CDC traps**	2.152	0.323	6.639	<0.0001
**October *CDC traps**	-2.933	0.431	-6.772	<0.0001
**Nov-Dec*CDC traps**	-4.148	0.443	-9.335	<0.0001

Table S5. Result of the best parsimonious of GLMM-1 of *Culex quinquefasciatus* female abundance in an inland village in Senegal. September, BG-sentinel trap, and indoor trapping location taken as reference levels. Number of observations (trap/house/night) = 240. Standard deviation of random effects = 0.35.

Variable	Coeff.	SE	z-value	$Pr(>\lvert z \rvert)$
**(Intercept)**	-0.525	0.257	-2.038	0.041
**Early-November**	2.063	0.244	8.463	<0.0001
**Mid- November**	2.513	0.243	10.349	<0.0001
**CDC traps**	1.055	0.299	3.523	<0.0001
**Outdoor**	1.329	0.18	7.380	<0.0001
**Early-November *CDC traps**	-1.941	0.349	-5.553	<0.0001
**Mid-November *CDC traps**	-2.677	0.354	-7.750	<0.0001
**CDC traps*Outdoor**	-1.432	0.276	-5.197	<0.0001

Table S6. Summary of the mean of *Culex quinquefasciatus* in the coastal and inland village predicted by GLMM-1. N= Number of traps/day in 9 and 10 houses in the coastal and inland village (4 night of sampling for three months). 95 % CI= 95 % of confidence interval.

Village	Months	Location	Traps (N)	Mean of *Culex quinquefasciatus*/p/n
Coastal	September	Not statistically significant in the GLMM-1	BG (9)	1,01 (95% CI 0,56/1,79)
			CDC (10)	8,70 (95 %CI 5,58/13,54)
	October	Not statistically significant in the GLMM-1	BG (9)	10,25 (95% CI 6,36/16,51)
			CDC (10)	4,96 (95% CI 2,98/7,39)
	Nov-Dec	Not statistically significant in the GLMM-1	BG (9)	13,71 (95% CI 8,55/22,00)
			CDC (10)	1,86 (95 % CI 1,15/3,02)
Inland	September	outdoor	BG (5)	2,23 (95 % CI 1,42/3,51)
			CDC (5)	1,53 (95% CI 0,94/2,49)
		indoor	BG (5)	0,59 (95 % CI 0,35/0,98)
			CDC (5)	1,69 (95% CI 1,09/2,63)
	Early-November	outdoor	BG (5)	17,58 (95% CI 11,81/26,17)
			CDC (5)	1,73 (95% CI 1,10/2,69)
		indoor	BG (5)	4,64 (95% CI 3,12/6,91)
			CDC (5)	1,91 (95% CI 1,19/3,06)
	Mid-November	outdoor	BG (5)	27,61 (95 %CI 18,85/ 40,45)
			CDC (5)	1,30 (95% CI 0,81-2,07)
		indoor	BG (5)	7,30 (95% CI 4,88/10,91)
			CDC (5)	1,43 (95% CI 0,89/2,32)

Table S7. Result of best parsimonious of GLM-1 model of *Anopheles gambiae* s.l. female abundance in the coastal village. September and BG traps as a reference level. Number of observations (trap/house/night) = 216 of sampling for three months by 5 CDC indoor/5 CDC outdoor and 4 BG indoor/4 BG outdoor (number of houses =9). Dispersion parameter (ϑ) =0.35

Variable	Coeff.	SE	z-value	$Pr(>\lvert z\rvert)$
**(Intercept)**	-0.247	0.306	-0.806	0.42
**October**	-1.855	0.388	-4.783	<0.0001
**Nov-Dec**	-2.275	0.423	-5.366	<0.0001
**CDC traps**	1.095	0.345	3.169	0.001

Table S8. Result of the best parsimonious of GLM-1 of *Anopheles gambiae* s.l. female abundance in an inland village in Senegal. September, BG-sentinel trap, indoor location taken as a reference levels. Number of observations (trap/house/night) = 240. Dispersion parameter (ϑ) = 0.41.

Variable	Coeff.	SE	z-value	$Pr(>\lvert z\rvert)$
**(Intercept)**	-2.479	0.646	-3.840	0.0001
**Outdoor**	0.368	0.582	0.633	0.526
**CDC traps**	3.088	0.734	4.209	<0.0001
**Early-November**	0.891	0.683	1.305	0.192
**Mid-November**	-0.333	0.841	-0.398	0.691
**CDC traps* Outdoor**	-1.627	0.742	-2.194	0.027
**CDC traps*Early-November**	-2.107	0.835	-2.525	0.011
**CDC traps*Mid-November**	-2.459	1.104	-2.226	0.025

Table S9. Summary of the mean of *Anopheles gambiae* s.l. in a coastal and inland village in Senegal predicted by GLM-1. N= Number traps/day in 9 and 10 houses in the coastal and inland village (4 night of sampling for three months). 95 % CI= 95 % of confidence interval.

Village	Months of collection	Trapping Location (indoor/outdoor)	Traps (N)	Mean of *An. gambiae (95% CI)*
Coastal	September	Not statistical significant in the GLMM-1	BG (9)	0,78(95 % CI 0,42/1,42)
			CDC (10)	2,33 (95%/ 1,40/3,89)
	October	Not statistical significant in the GLMM-1	BG (9)	0,12 (95 % 0,05/0,26)
			CDC (10)	0,36 (95% CI 0,19/0,69)
	Nov-Dec	Not statistical significant in the GLMM-1	BG (9)	0,08 (95 % CI 0,03/0,18)
			CDC (10)	0,23 (95 % CI 0,11/0,49)
Inland	September	outdoor	BG (5)	0,12 (95 %CI 0,03/0,39)
			CDC (5)	0,52 (95 % CI 0,23/1,14)
		indoor	BG (5)	0,08 (95% CI 0,02/0,29)
			CDC (5)	1,83 (95% CI 0,92/3,63)
	Early-November	outdoor	BG (5)	0,29 (95 %CI 0,11/0,75)
			CDC (5)	0,15 (95% CI 0,05/0,40)
		indoor	BG (5)	0,20 (95% CI 0,07/0,56)
			CDC (5)	0,54 (95 % CI 0,24/1,20)
	Mid-November	outdoor	BG (5)	0,08 (95% CI 0,02/0,32)
			CDC (5)	0,03 (95% CI 0,007/0,0,13)
		indoor	BG (5)	0,05 (95% CI 0,01/0,24)
			CDC (5)	0,11 (95% CI 0,03/0,11)

Table S10. Result of the best parsimonious of GLM-3 of *Anopheles arabiensis, An. gambiae* and *An. coluzzii* probability in a coastal village in Senegal. September taken reference level. Number of observations (trap/house/night) = 216.

Species	Variable	Coeff.	SE	z-value	Pr(>|z|)
	**(Intercept)**	-0.955	0.262	-3.632	0.0002
An. arabiensis	**October**	-1.556	0.404	-1.379	0.16
	**Nov-Dec**	-1.640	0.533	-3.076	0.02
	**(Intercept)**	-1.713	0.328	-5.229	<0.0001
An. gambiae ss	**October**	-1.842	0.787	-2.337	0.018
	**Nov-Dec**	-2.549	1.059	-2.408	0.015
	**(Intercept)**	-1.421	0.298	-4.733	<0.0001
An. coluzzii	**October**	-2.841	1.049	-2.706	0.006
	**Nov-Dec**	-2.134	0.776	-2.748	0.006

Table S11. Result of the best parsimonious of GLM-3 of *Anopheles arabiensis* and *An. gambiae* probability in a inland village in Senegal. BG taken reference level. Number of observations (trap/house/night) = 240.

Species	Variable	Coeff.	SE	z-value	Pr(>|z|)
An. arabiensis	**(Intercept)**	-2.639	0.366	-7.211	<0.0001
	**CDC traps**	0.968	0.442	2.185	0.029
An. gambiae ss	**(Intercept)**	-3.663	0.584	-6.266	<0.0001
	**CDC traps**	1.632	0.649	2.521	0.017

Table S12. Result of GLM-2 of the mean of *Culex quinquefasciatus*/trap/night in a coastal village in Senegal. September collections taken as reference level. Number of observations (trap/house/night) = 215 Dispersion parameter (ϑ) = 0.47.

Variable	Coeff.	SE	z-value	Pr(>|z|)
**(Intercept)**	2.300	0.121	18.949	<0.0001
**CDC Traps**	-1.750	0.182	-9.572	<0.0001

Table S13. Result of GLM-2 of the mean of *Culex quinquefasciatus*/trap/night in an inland village in Senegal. September collection is taken as a reference level. Number of observations (trap/house/night) = 240. Dispersion parameter (ϑ) = 0.71.

Variable	Coeff.	SE	z-value	Pr(>|z|)
**(Intercept)**	2.300	0.121	18.949	<0.0001
**CDC Traps**	-1.750	0.182	-9.572	<0.0001

Table S14. Result of GLM-2 of mean *Anopheles gambiae* s.l. females/trap/night in a coastal village in Senegal. BG-Sentinel taken as reference level. Number of observations (trap/house/night) = 216. Dispersion parameter (ϑ) =0.19.

Variable	Coeff.	SE	z-value	Pr(>|z|)
**(Intercept)**	-1.099	0.289	-3.801	<0.0001
**CDC Traps**	1.065	0.366	2.909	0.003

Table S15. Result of GLM-2 of mean *Anopheles gambiae* s.l. females/trap/night in an inland village in Senegal. September taken as reference level. Number of observations (trap/house/night) = 240. Dispersion parameter (ϑ) =0.18

Variable	Coeff.	SE	z-value	Pr(>|z|)
**(Intercept)**	-1.954	0.323	-6.036	<0.0001
**CDC Traps**	1.371	0.407	3.369	<0.0001

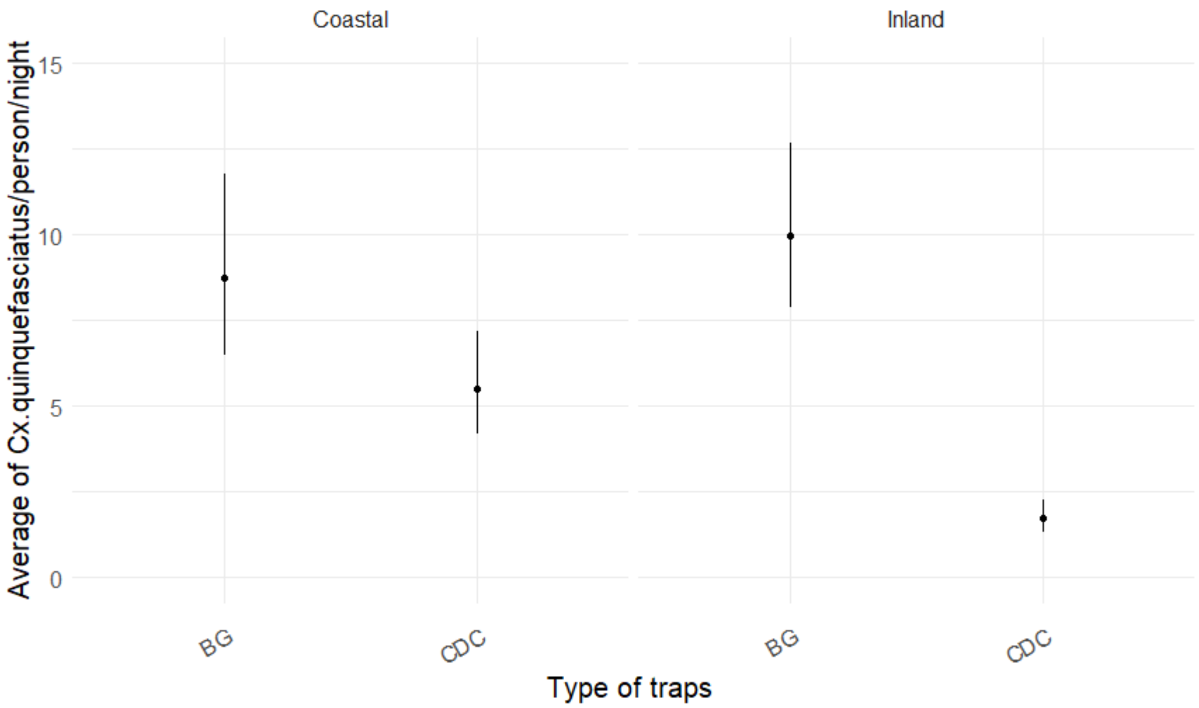

Figure S1. Average number of *Culex quinquefasciatus* host-seeking females/trap/night collected by BG-sentinel and CDC-light traps in a coastal (Left) and in an inland (Right) village in Senegal as estimated by GLM-2. Dots=average number mosquito females/person/night. Black vertical lines=95% confidence intervals.

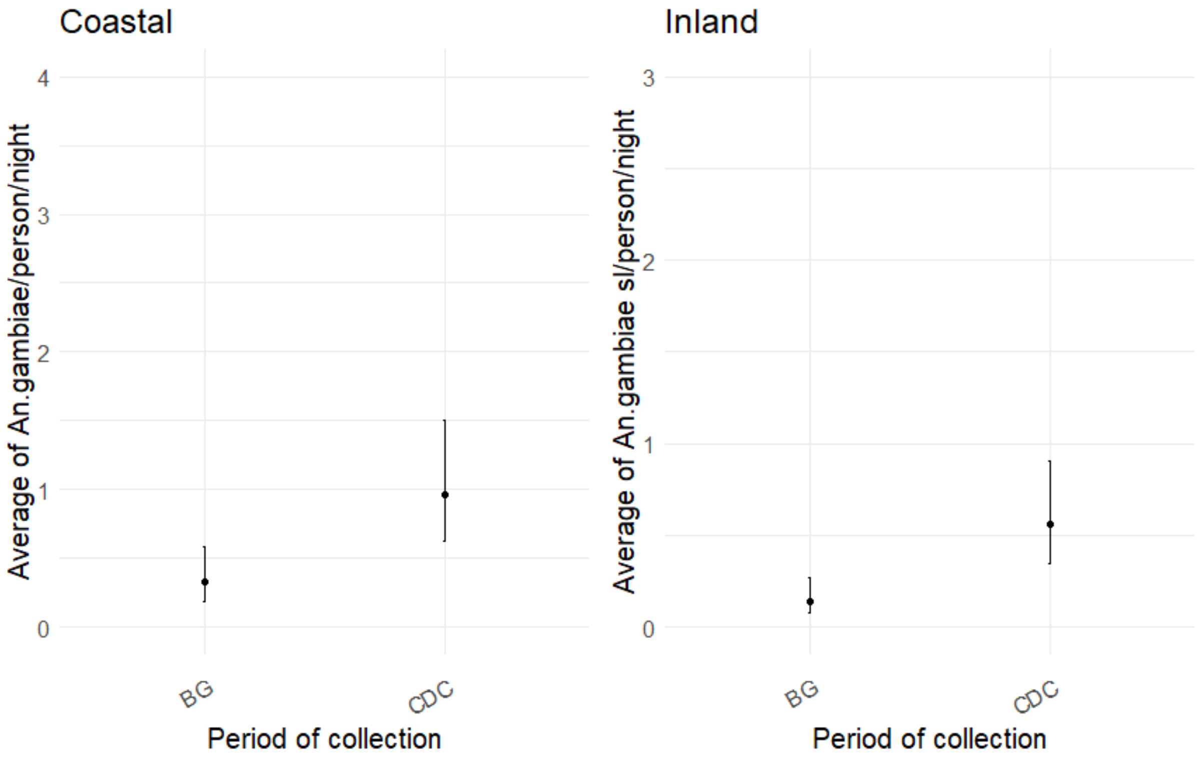

Figure S2. Average number of *Anopheles gambiae* s.l. host-seeking females/trap/night collected by BG-sentinel and CDC-light traps in a coastal (Left) and in an inland (Right) village in Senegal, as estimated by GLM-1. Dots=average number mosquito females/trap/night. Vertical lines=95% confidence intervals.
